# Outcomes of Surgical Versus Conservative Management in Stanford Type a Aortic Dissection: A Single-Center Retrospective Study

**DOI:** 10.3390/life15030462

**Published:** 2025-03-14

**Authors:** Irina-Anca Eremia, Mihnea-Ioan-Gabriel Popa, Cătălin-Alexandru Anghel, Teodora-Adriana Stroe, Eduard-Alexandru Eremia, Andreea Nicoleta Marinescu, Remus Iulian Nica, Silvia Nica

**Affiliations:** 1 Department of Family Medicine III, Carol Davila University of Medicine and Pharmacy, 050474 Bucharest, Romania; irina.eremia@umfcd.ro; 2 Emergency Department, Emergency University Hospital Bucharest, 050098 Bucharest, Romania; catalin-alexandru.anghel@rez.umfcd.ro; 3 Department of Orthopedics and Traumatology, Carol Davila University of Medicine and Pharmacy, 050474 Bucharest, Romania; 4 Department of Orthopedics and Traumatology, Emergency University Hospital Bucharest, 050098 Bucharest, Romania; 5 Faculty of Medicine, Carol Davila University of Medicine and Pharmacy, 050474 Bucharest, Romania; teodora-adriana.stroe0721@stud.umfcd.ro; 6 Faculty of Medicine, Ludwig Maximilian University of Munich, 80336 Munich, Germany; ere.edu01@gmail.com; 7 Department of Radiology and Medical Imaging, Carol Davila University of Medicine and Pharmacy, 050474 Bucharest, Romania; andreea_marinescu@umfcd.ro; 8 Department of Radiology and Medical Imaging, Emergency University Hospital Bucharest, 050098 Bucharest, Romania; 9 Discipline of General Surgery, Carol Davila University of Medicine and Pharmacy, 050474 Bucharest, Romania; remus.nica@umfcd.ro; 10 Surgery Department, Central Military Emergency University Hospital “Dr. Carol Davila”, 010825 Bucharest, Romania; 11 Department of Emergency and First Aid, Emergency University Hospital Bucharest, Carol Davila University of Medicine and Pharmacy, 050474 Bucharest, Romania; silvia.nica@umfcd.ro

**Keywords:** postoperative complications, in-hospital mortality, surgical management, Stanford Type A, acute aortic dissection

## Abstract

Acute aortic dissection (AAD) is a critical cardiovascular emergency marked by the rupture of the aortic intima, resulting in blood infiltration into the media and the formation of a false lumen. AAD incidence varies by area, emphasizing the need for better diagnostics and epidemiological investigations. Bucharest University Emergency Hospital’s Emergency Department conducted this retrospective cohort analysis from May 2021 to May 2023. We examined 26 Stanford Type A aortic dissection patients to establish in-hospital mortality and one-year survival rates. The primary objective was to analyze demographic, clinical, and paraclinical factors and their impact on patient outcomes. A total of 57.7% of the study group was male and had a mean age of 58.2 years, and 69.2% of patients had hypertension, indicating its importance as a risk factor. Acute chest discomfort was reported by 53.8%, neurological problems by 30.8%, and syncope or hypotension by 42.3%. CT angiography and transthoracic echocardiogram (TTE) confirmed the diagnosis and assessed dissection severity. Pericardial effusion (19.2%) and moderate to severe aortic regurgitation (26.9%) were notable. Management varied by dissection intensity and location. Emergency surgery was performed in 61.5% of patients within 24 h of diagnosis, resulting in a 12.5% in-hospital death rate. Conservatively managed patients had a 60.0% in-hospital death rate. Timely intervention is crucial, since the surgical cohort had an 87.5% one-year survival rate compared to 30% for the conservatively managed cohort. Acute renal damage (25%), protracted mechanical ventilation (31.3%), and advanced supportive care infections were postoperative sequelae. Conservative care exacerbated visceral ischemia (20%) and heart failure (10%). Advanced age and hypotension upon admission were independent mortality predictors, emphasizing the need for early risk assessment and personalized treatment. Multimodal imaging, timely surgical referral, and excellent postoperative care improve AAD outcomes, according to this study.

## 1. Introduction

Aortic dissection is a severe cardiovascular condition characterized by blood flow disrupting the intima and penetrating the media of the aortic wall. This process divides the wall into two strata, resulting in a false lumen that undermines the structural integrity of the aorta. Dissection may result in significant consequences, contingent upon its location and severity, such as aortic rupture, organ ischemia from arterial branch involvement, acute valvular insufficiency, or cardiac tamponade [1].

The global incidence of aortic dissection varies according to geographic locations and the availability of diagnostic resources. The frequency in Europe varies from 2.53 to 7.2 cases per 100,000 people, whereas in North America, specifically Canada and the USA, it ranges from 4.6 to 10 cases per 100,000 population. Japan has a high incidence in Asia, with up to 17.6 instances per 100,000, while China and Korea report lower rates. The increased occurrences in Oceania and the lack of data from Africa underscore the necessity of epidemiological investigations [2].

The clinical manifestation of aortic dissection is heterogeneous and varies based on the severity of the dissection and the cardiovascular tissues affected. Patients typically exhibit intense anterior or posterior chest pain, characterized as a “tearing sensation”, which radiates as the dissection advances. This may or may not correlate with further symptoms, including pulse deficits, heart murmurs, focal neurological impairments, hypotension, or syncope [3,4,5,6]. The complexity of this disorder necessitates differential diagnosis in emergency settings to distinguish it from other conditions marked by anterior or posterior chest discomfort, pulse abnormalities, and neurological manifestations. Vascular pathologies encompass acute coronary syndrome, pneumothorax, pulmonary embolism, pericarditis, esophageal rupture, and peptic ulcer. Furthermore, if the dissection encompasses the descending aorta and progresses distally, the pain may spread along the spinal column, resembling the signs and symptoms of renal colic [4,7,8].

**Figure 1:**
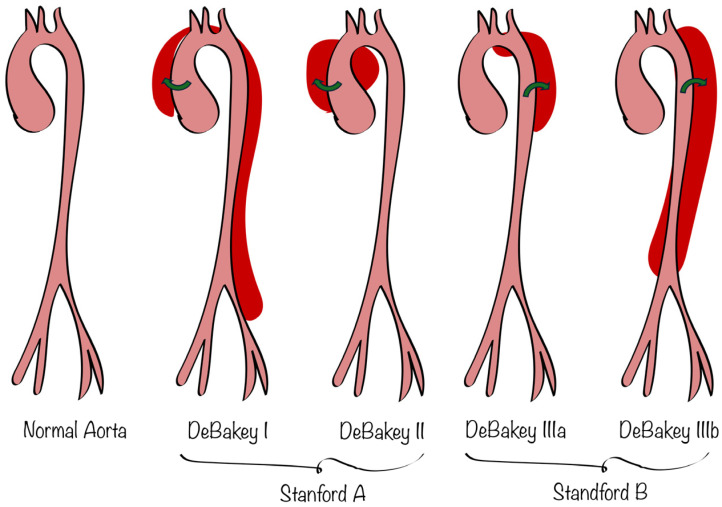
Stanford and DeBakey classification of aortic dissection (graphics program used: Adobe Inc., San Jose, CA, USA, Adobe Illustrator 2019, Version 23.1.0. Adobe Illustrator. Retrieved from https://adobe.com/products/illustrator, accessed on 1 February 2025).

The Stanford and DeBakey Classification (Figure 1) are crucial for identifying and managing aortic dissection, since they elucidate the dissection’s location and degree, thus furnishing vital information for treatment considerations. Type A dissections (Stanford) and Types I and II (DeBakey) present a considerable risk of rupture or cardiac tamponade, while Type B dissections (Stanford) and Type III (DeBakey) may be managed conservatively, with intervention limited to complications like visceral ischemia or rupture. These distinctions directly enhance patient survival through timely and tailored therapeutic interventions [9].

The immediate therapy of aortic dissection necessitates prompt action to stabilize the patient and reduce the likelihood of life-threatening complications. After confirming the diagnosis, immediate consultation with a cardiovascular surgeon is essential. In the absence of surgical services, the patient must be expeditiously moved to a specialized facility. Immediate resuscitation, blood pressure management, and heart rate decrease via intravenous injection of beta-blockers or calcium channel blockers are paramount. These methods aim to reduce tension on the aortic wall and lower the risk of rupture [9,10].

The choice of therapeutic approach depends on the dissection’s location and severity, ranging from intensive medical management to emergency surgical intervention. Type A dissections (Stanford) generally necessitate immediate surgical intervention to repair or replace the compromised segment of the aorta [9,11,12]. In contrast, Type B dissections (Stanford), affecting solely the descending aorta, may be managed conservatively at the outset. This necessitates rigorous regulation of blood pressure and heart rate to avert problems. Patients are meticulously observed for indications of visceral ischemia, aortic rupture, or renal failure, which may necessitate surgical intervention [9,13,14]. This study investigates the demographic, clinical, and paraclinical data of patients with Stanford Type A acute aortic dissection (AAD) and examines factors influencing mortality and long-term outcomes.

## 2. Materials and Methods

### 2.1. Study Design and Setting

This research was a retrospective observational cohort study carried out at the Emergency Department of Bucharest University Hospital from May 2021 to May 2023. The study aimed to evaluate the demographic, clinical, and paraclinical characteristics of patients diagnosed with AAD, particularly Stanford Type A dissections, and to identify factors associated with in-hospital mortality and one-year overall survival. Ethical approval was obtained from the Local Ethics Committee of Bucharest University Hospital (approval code: 63334, 18 October 2024), and all patient data were anonymized according to national data protection regulations.

### 2.2. Patient Population

The study population included patients aged 18 years and older, who were admitted with a confirmed diagnosis of AAD. Patients included in the study were exclusively those diagnosed with Stanford Type A dissections, as determined by initial clinical evaluation and confirmed through imaging techniques such as angio-CT or echocardiography. Exclusion criteria comprised incomplete medical records, absence of essential clinical data, or an unverified diagnosis through imaging. A total of 26 patients were included in the analysis after applying the inclusion/exclusion criteria. Demographic data, including age, sex, body mass index (BMI), and pertinent clinical variables such as history of hypertension (HTA), diabetes mellitus, smoking status, pheochromocytoma, and drug use, were gathered. The predisposing factors for Stanford Type A AAD include chronic hypertension; atherosclerosis; genetic conditions such as Marfan syndrome, Loeys–Dietz syndrome, and Ehlers–Danlos syndrome; and bicuspid aortic valve disease. Additionally, acute triggers such as sudden hemodynamic stress, intense physical exertion, and emotional distress can contribute to the onset of the dissection. In our cohort, hypertension was the most frequently observed risk factor (69.2%), followed by smoking (38.5%) and diabetes mellitus (26.9%). These factors highlight the importance of early risk assessment and targeted preventive strategies.

### 2.3. Data Collection

Data collection was conducted retrospectively utilizing both paper-based medical records and the electronic health record (EHR) system of the hospital. The collected variables comprised demographic information (age, sex, BMI), clinical risk factors, and essential paraclinical measurements, including blood pressure measurements, both systolic and diastolic, recorded at the time of admission; assessment of peripheral pulse presence and associated neurological deficits, categorized as either absent or present; characteristics of chest pain and episodes of syncope; and laboratory values encompassing full blood count, kidney function indicators (urea, creatinine), and cardiac biomarkers, including creatine kinase (CK), CK-MB, and high-sensitivity cardiac troponin I (hs-cTnI). Imaging data were acquired through transthoracic echocardiography (TTE) and thoracic computed tomography angiography (CTA).

**Figure 2:**
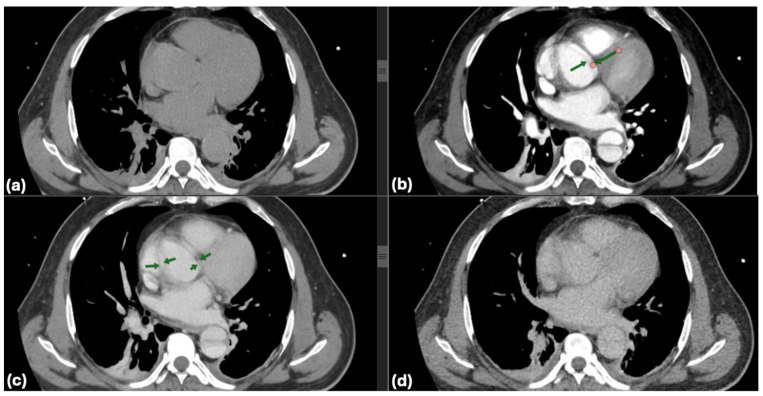
Artifacts in non-gated computed tomography: pulsation effects in aortic imaging: common artifacts from pulsation in non-gated computed tomography (CT) imaging are highlighted with green arrows (**b**,**c**). These artifacts can obscure critical details of the aortic wall and luminal structure, potentially complicating the assessment of aortic dissections (**a**,**d**). Accurate interpretation requires recognizing and mitigating such limitations.

All patients received TTE upon admission. The assessment was conducted on left ventricular ejection fraction (LVEF), the presence of pericardial effusion, and wall motion abnormalities. The dimensions of the aorta and the presence of aortic valve pathology were recorded. CTA was conducted on all patients to verify the diagnosis of dissection, determine the extent of dissection (including the ascending aorta, aortic arch, and descending aorta), and assess for complications such as periaortic hematoma, rupture, or involvement of visceral arteries. Scans utilized a 128-slice CT scanner with contrast enhancement to delineate both true and false lumens. One of the critical aspects of evaluating aortic pathologies is the accurate visualization of the aortic lumen and wall structures through CT imaging. However, non-gated CT scans are susceptible to pulsation artifacts, which can obscure essential details and lead to diagnostic challenges. Common pulsation artifacts observed in this study are illustrated in Figure 2.

The management of patients with Stanford Type A dissection is predominantly surgical. Emergency surgery was conducted within 24 h of diagnosis for patients exhibiting hemodynamic instability, indications of aortic rupture, or rapidly deteriorating symptoms.

#### 2.3.1. Surgical Intervention

In cases of Type A dissections, a median sternotomy was performed, subsequently leading to the replacement of the ascending aorta using a Dacron graft (ZOTEC HGA, Esslingen am Neckar, Germany). In instances where the dissection involved the aortic arch, the procedure was modified to incorporate arch replacement. Cardiopulmonary bypass was commenced with hypothermic circulatory arrest as required.

#### 2.3.2. Medical Management

Patients deemed unsuitable for surgery, whether due to comorbidities or personal refusal, were managed through medical interventions. Intravenous beta-blockers were administered to decrease heart rate and systolic blood pressure, with the objective of reducing shear stress on the aortic wall. Blood pressure targets were established as below 120 mmHg systolic. Furthermore, vasodilators and calcium channel blockers were employed as supplementary agents when beta-blockade alone proved inadequate.

#### 2.3.3. Postoperative Care

Patients were transferred to the intensive care unit (ICU) following surgery, where hemodynamic monitoring, respiratory support, and renal replacement therapy were implemented as required. Postoperative complications, such as acute kidney injury (AKI), respiratory failure, and sepsis, were closely monitored in patients. Long-term follow-up involved regular CT scans to evaluate aortic morphology and integrity.

In our cohort, preoperative management followed a standardized protocol, with adjustments tailored to individual clinical presentations. Among the 26 patients included, 12 (46.1%) required immediate stabilization in the emergency department due to severe hypotension (systolic BP < 90 mmHg) and signs of end-organ hypoperfusion. Pharmacological intervention with intravenous beta-blockers (esmolol) was administered in all cases to reduce aortic wall shear stress, while sodium nitroprusside was added in eight patients (30.8%) to achieve target systolic pressures below 120 mmHg. Fluid resuscitation was initiated in six patients (23.1%) who presented with concurrent hypovolemia, while inotropic support (dobutamine) was necessary for three critically unstable patients (11.5%). Imaging findings from CTA played a central role in decision-making; in four patients, significant pericardial effusion and mediastinal hematoma prompted immediate surgical prioritization. Intraoperatively, nine cases (34.6%) required total aortic arch replacement due to extensive dissection involving the brachiocephalic trunk and left subclavian artery. Additionally, hypothermic circulatory arrest was employed in seven patients (26.9%) to enable precise graft placement and minimize ischemic injury. Postoperatively, intensive care management included continuous renal replacement therapy (CRRT) in four patients (15.4%) due to AKI and prolonged mechanical ventilation (>48 h) in five patients (19.2%). Early mobilization was achieved in 10 patients (38.5%) within 48 h post-surgery, while targeted respiratory physiotherapy reduced pulmonary complications in six patients (23.1%). These findings emphasize that outcomes in Stanford Type A dissections are profoundly influenced not only by surgical intervention but also by the precision of preoperative stabilization, intraoperative decision-making, and tailored postoperative care.

### 2.4. Outcome Measures

This study evaluated in-hospital mortality and one-year survival as primary outcomes, with secondary outcomes including hospitalization duration and post-surgical complications. Outcome data were collected from patient follow-up records, including clinic visits, hospital readmissions, and, where relevant, phone interviews with surviving patients or their families.

### 2.5. Stastical Analysis

Statistical analyses were conducted utilizing Statistical Package for the Social Sciences (IBM SPSS Statistics for Windows, IBM Corp., Version 26.0, Armonk, NY, USA). The dataset was cleaned and prepared before analysis, ensuring the exclusion of missing or incomplete cases. Descriptive statistics summarized the baseline characteristics of the patients. Continuous variables, including age, BMI, and duration of hospitalization, were presented as means ± standard deviations. Categorical variables, such as survival status and presence of complications, were reported as percentages.

#### 2.5.1. Group Comparisons

Continuous variables were assessed using the independent samples *t*-test, whereas categorical variables were evaluated through the Chi-square test or Fisher’s exact test as applicable. Survival analysis involved the generation of Kaplan–Meier survival curves to assess one-year survival rates, with subgroup differences (e.g., surgical versus medical management) analyzed using the log-rank test.

#### 2.5.2. Models of Regression

Logistic regression models were utilized to determine clinical and demographic factors linked to in-hospital mortality and extended hospitalization. Multivariate analyses utilized the Cox proportional hazards model to determine independent predictors of mortality.

#### 2.5.3. Statistical Significance

A *p*-value of less than 0.05 was deemed statistically significant for all analyses.

## 3. Results

**Figure 3:**
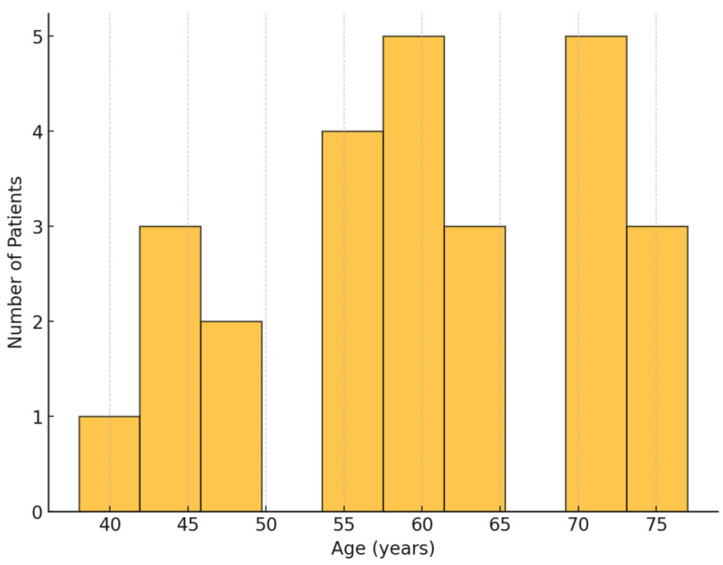
Age distribution: this graph depicts the age distribution of patients participating in the study. The bulk of patients are situated among the 51–60 and 61–70 age brackets, indicating a greater frequency of this pathology among older adults.

The study included 26 patients, with a mean age of 58.2 ± 8.7 years (Figure 3). The cohort included 15 males (57.7%) and 11 females (42.3%), indicating a marginally higher incidence of AAD among males. Most patients (69.2%) had a history of hypertension, a recognized risk factor for AAD. Diabetes mellitus was present in 26.9% of patients, while 38.5% had a history of smoking. The average BMI was 26.8 ± 3.2 kg/m^2^, showing no significant difference between genders. Notably, none of the patients in the study received a confirmed diagnosis of pheochromocytoma; however, 11.5% reported substance abuse, predominantly involving stimulants. A significant proportion of patients (53.8%) exhibited tearing chest pain, a hallmark symptom of dissection. Additionally, 30.8% presented with neurological complications, primarily manifesting as transient ischemic attacks (TIAs) and stroke. Management included early neurological assessment, targeted blood pressure control, and, in select cases, imaging-guided thrombolysis.

**Figure 4:**
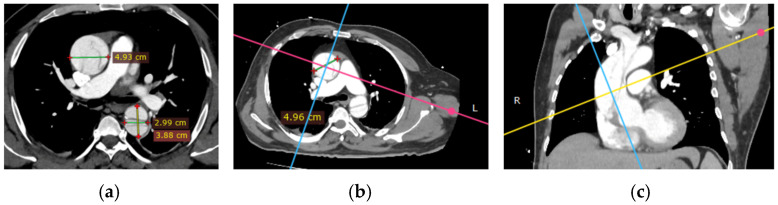
Radiological measurements of the aortic lumen using perpendicular sections in a patient with aortic pathology: (**a**) axial view showing key diameters at different levels: ascending aorta (4.93 cm), descending aorta (2.99 cm), and distal descending aorta (3.88 cm; (**b**) the cross-sectional plane used for accurate measurements (4.96 cm); (**c**) coronal reconstruction illustrating the orientation of the imaging planes relative to the aortic arch and descending aorta, ensuring perpendicular alignment for precise dimensional assessment. These measurements are critical for diagnosing and monitoring the progression of aortic diseases and planning surgical or interventional management.

Imaging is essential for the diagnostic confirmation of aortic dissection. All patients received thoracic CTA and TTE. In the radiological evaluation of patients with aortic pathologies, precise measurements of the aortic lumen play a crucial role in assessing disease severity and planning interventions. Using true perpendicular sections, accurate diameters of the ascending and descending aorta were obtained. The following images (Figure 4) demonstrate this approach and provide representative measurements for reference.

**Figure 5:**
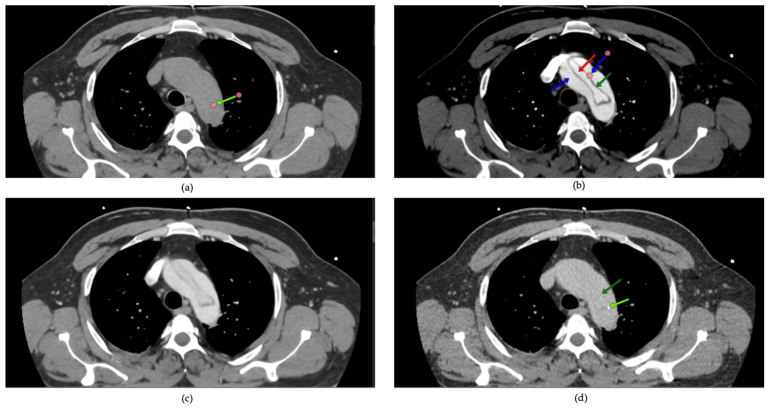
Multiphase CT imaging of Stanford Type A aortic dissection: true and false lumen analysis; (**a**–**d**): axial CT images demonstrating characteristic findings of Stanford type A aortic dissection. Dark green arrow: intimal flap, marking the division between the true and false lumen; red arrow: true aortic lumen, compressed and reduced in size due to the higher pressure within the false lumen; blue arrow: false aortic lumen, which surrounds the true lumen and shows delayed opacification (arterial phase visible (**b**) versus venous phase in (**c**)); light green arrow: intimal calcifications within the aortic wall.

CTA indicated that all cases were associated with Stanford Type A dissections. In 23.1% of patients, dissection extended to the aortic arch, whereas 38.5% exhibited involvement of the descending aorta. Echocardiography results revealed that 26.9% of patients exhibited moderate to severe aortic regurgitation, while 19.2% showed pericardial effusion, thereby heightening the necessity for surgical intervention. The mean LVEF was 56.7%, suggesting that cardiac function was preserved in the majority of patients prior to surgery. Radiological evaluation of Stanford Type A aortic dissections provides essential insights into the pathophysiology and anatomy of the condition. Axial CT sections are particularly valuable in delineating the true and false lumens, as well as the intimal flap that separates them. Figure 5 highlights these features, demonstrating the compressed true lumen and the delayed opacification of the false lumen in the venous phase compared to the arterial phase. Additional findings, such as intimal calcifications, further contribute to a comprehensive understanding of the imaging characteristics of this pathology.

Among the 26 patients, 16 (61.5%) received emergency surgery within 24 h of diagnosis, whereas the remaining 10 patients were managed conservatively due to surgical contraindications or refusal by the patient or family. In the surgically treated cohort, 81.3% achieved successful aortic repair or replacement utilizing Dacron grafts, accompanied by an in-hospital mortality rate of 12.5%. Patients managed conservatively exhibited a markedly elevated in-hospital mortality rate of 60.0% (*p* < 0.05), underscoring the essential role of early surgical intervention in Stanford Type A dissections. The mean duration of hospitalization for survivors was 22.5 ± 12.3 days. Patients who underwent surgery experienced shorter hospital stays, averaging 18.5 ± 10.7 days, in contrast to those receiving medical management, who had an average stay of 28.7 ± 13.5 days (*p* < 0.05).

To further illustrate the relationship between clinical parameters and short-term outcomes, Table 1 presents patient characteristics stratified by 30-day survival. Age, systolic blood pressure, renal function, left ventricular ejection fraction, pericardial effusion, and neurological deficits were significantly associated with early mortality (*p* < 0.05). Time from symptom onset to surgery was also a critical determinant, with delayed intervention correlating with worse outcomes (*p* = 0.03). Left ventricular ejection fraction (LVEF) was significantly lower in non-survivors (49.2 ± 5.8%) compared to survivors (57.8 ± 6.2%, *p* = 0.01), suggesting a potential role of baseline cardiac function in influencing postoperative outcomes. Mean CPB time was slightly longer in non-survivors (105.6 ± 18.4 min) compared to survivors (92.3 ± 15.6 min, *p* = 0.06). Additionally, time from symptom onset to surgery was significantly delayed in non-survivors (16.8 ± 5.6 h vs. 10.5 ± 3.2 h, *p* = 0.03), reinforcing the need for rapid surgical intervention. Preoperative complications were more frequent in non-survivors (75.0%) compared to survivors (38.9%, *p* = 0.02), and the incidence of postoperative complications was also significantly higher (50.0% vs. 22.2%, *p* = 0.04).

**Figure 6:**
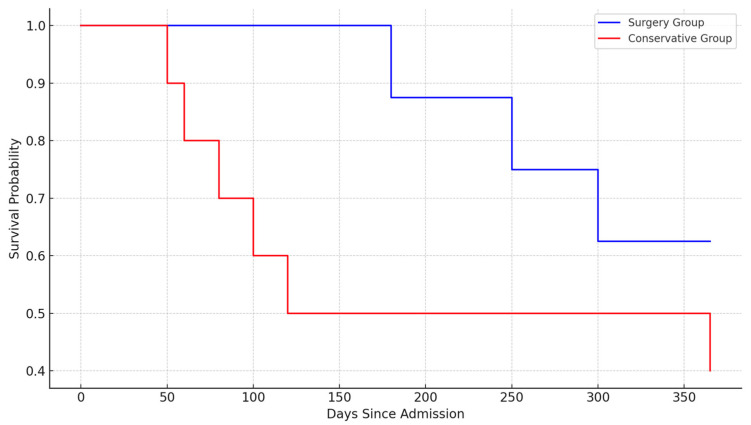
Kaplan–Maier survival curve demonstrating one-year survival comparison between surgical and conservative management approaches.

The one-year survival rate for the entire cohort was 61.5%. The one-year survival rate among patients who underwent surgery was 87.5%, significantly higher than the 30% observed in the conservatively managed group (*p* < 0.01). Kaplan–Meier survival analysis indicated a distinct survival benefit for patients undergoing surgical treatment, with log-rank testing validating a statistically significant difference (*p* < 0.01) (Figure 6).

**Figure 7:**
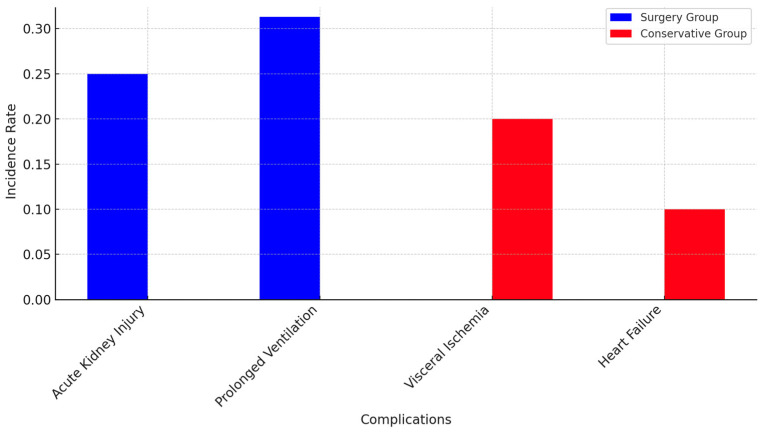
Incidence of postoperative and conservative management complications in surgical and conservative groups.

Postoperative complications were observed in 25% of patients, specifically AKI, necessitating CRRT (Figure 7). Furthermore, prolonged mechanical ventilation lasting over 48 h was required for 31.3% of surgical patients. Conversely, patients receiving conservative management exhibited a greater incidence of visceral ischemia (20%) and acute heart failure (10%). Independent *t*-tests were performed to compare continuous variables, including age, BMI, and hospitalization duration, between the surgical and conservative management groups. The mean age in the conservative group was 61.2 ± 9.4 years, while in the surgical group, it was 55.6 ± 7.2 years. However, the difference was not statistically significant (*p* = 0.08), indicating that advanced age and admission hypotension as independent predictors of in-hospital mortality are not a key factor in treatment decisions. Patients in the conservative group experienced a significantly longer hospitalization duration (28.7 ± 13.5 days) than those in the surgical group (18.5 ± 10.7 days, *p* = 0.02), indicating a more favorable recovery profile for surgically managed patients. The Chi-square test was employed to analyze categorical variables, such as survival status and complications. The findings indicated a markedly elevated occurrence of AKI in the surgical cohort (25%) relative to the conservative cohort (0%, *p* = 0.03). This finding correlates with the postoperative stress and invasive characteristics of the procedures, which may contribute to kidney dysfunction during the immediate recovery phase.

**Figure 8:**
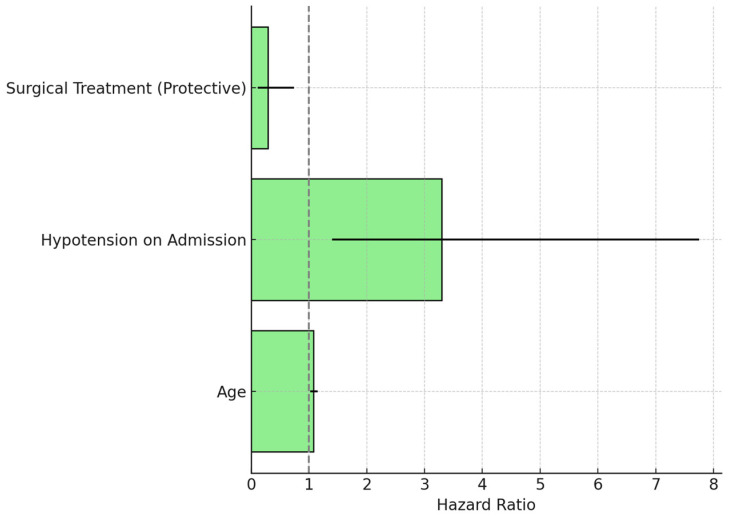
Hazard ratios for predictors of one-year mortality in Stanford Type A dissections.

Univariate analysis indicated that factors significantly correlated with elevated in-hospital mortality were advanced age (*p* = 0.03), hypotension upon admission (*p* = 0.02), and the presence of neurological deficits (*p* = 0.01). Logistic regression identified these factors as independent predictors, yielding odds ratios of 1.12 (95% CI 1.02–1.24) for age and 3.45 (95% CI 1.28–9.25) for hypotension (Figure 8). Logistic regression models were utilized to determine independent predictors of in-hospital mortality. The analysis indicated that advanced age (OR: 1.12, 95% CI: 1.02–1.24, *p* = 0.03) and hypotension at admission (OR: 3.45, 95% CI: 1.28–9.25, *p* = 0.01) were significant predictors of mortality, independent of other factors. The identified factors indicate a heightened risk of adverse outcomes in elderly patients and those exhibiting hemodynamic instability at presentation. A multivariate Cox proportional hazards model identified predictors of one-year mortality. The results of these analyses are summarized in Table 2. Advanced age and admission hypotension were significantly associated with in-hospital mortality, while surgical treatment was strongly predictive of improved one-year survival. The model incorporated variables such as age, sex, admission hypotension, neurological deficits, and treatment modality (surgical versus conservative). The analysis indicated that admission hypotension and treatment modality were the primary predictors of one-year survival.

Hypotension was identified as a significant predictor of one-year mortality, exhibiting a hazard ratio (HR) of 3.30 (95% CI: 1.40–7.75, *p* = 0.005). Patients exhibiting hypotension demonstrated a threefold elevation in the risk of mortality within one year relative to those maintaining stable hemodynamics.

The type of treatment (surgical versus conservative) emerged as a significant predictor, with patients undergoing surgery exhibiting a markedly reduced risk of one-year mortality (HR: 0.29, 95% CI: 0.11–0.74, *p* = 0.01). This finding highlights the critical importance of early surgical intervention in the management of Stanford Type A dissections, as surgery significantly decreases the risk of long-term mortality.

To further analyze the impact of surgical timing on patient outcomes, we categorized patients into early-surgery (≤12 h) and late-surgery (>12 h) groups. Table 3 summarizes the results. Patients who underwent surgery within the first 12 h had a significantly lower 30-day mortality (10.0%) compared to those with delayed surgery (>12 h), where mortality was 50.0% (*p* = 0.02). Additionally, early surgery was associated with a higher one-year survival rate (90.0% vs. 50.0%, *p* = 0.03). These findings emphasize the critical importance of prompt surgical intervention in Stanford Type A AAD.

## 4. Discussion

The management of AAD, especially Stanford Type A dissections, continues to pose a significant problem in cardiovascular medicine. Our research corroborates current literature, highlighting the significance of early diagnosis and timely surgical intervention to reduce mortality and enhance long-term results. The cohort’s mean age of 58.2 years corresponds with research indicating that AAD predominantly impacts adults in their 60s. Likewise, the male predominance (57.7%) supports previous data indicating that AAD is more prevalent in males, but females typically appear at a later stage with potentially poorer prognoses [14,15,16].

Hypertension, identified as a critical risk factor, was observed in 69.2% of our group, aligning with the International Registry of Acute Aortic Dissection (IRAD), which reported a prevalence of 72%. Genetic predispositions and environmental stressors, including poorly managed hypertension and obesity, have been recognized as contributing causes, highlighting the necessity for targeted preventative interventions [17,18]. Variability in clinical presentation was seen, with merely 53.8% of patients experiencing typical ripping chest pain, underscoring the diagnostic intricacy of AAD. The presence of neurological deficits in 30.8% and hypotension/syncope (42.3%) in patients reflects the systemic nature of aortic dissections. These neurological complications included TIAs, cerebrovascular accidents (stroke), and episodes of altered consciousness, often resulting from impaired cerebral perfusion caused by malperfusion syndromes or hypotensive events. Immediate neurological assessment, coupled with strict blood pressure management and imaging-guided monitoring of cerebral perfusion, played a pivotal role in preventing further neurological deterioration. Early recognition and targeted intervention were critical in minimizing long-term neurological morbidity. However, patients presenting with neurological symptoms experienced prolonged ICU stays and a higher incidence of postoperative complications, underscoring their significant prognostic impact. These findings corroborate the existing literature, which emphasizes the importance of incorporating AAD in the differential diagnosis of patients presenting with neurological symptoms, even in less common clinical scenarios [15,19].

**Figure 9:**
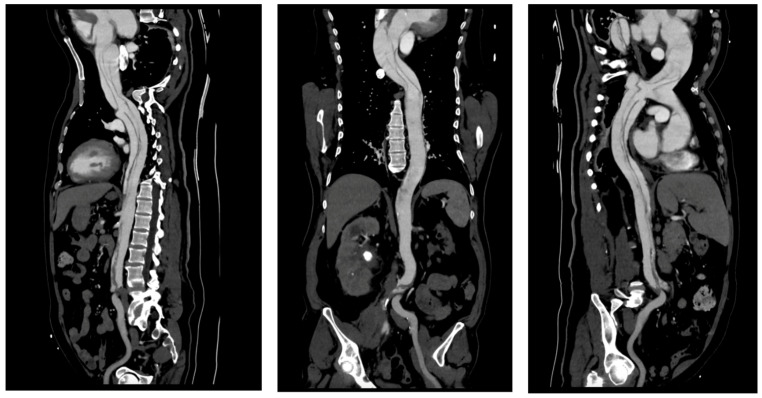
CT imaging of Stanford Type A aortic dissection: true and false lumen analysis—sagittal views: The sagittal images highlight an extensive dissection flap extending from the ascending aorta through the aortic arch and into the descending thoracic and abdominal aorta. The false lumen is clearly visible, with signs of thrombosis involving both the right and left common iliac arteries. These findings indicated a high risk of malperfusion and were pivotal in the decision to proceed with emergency surgical intervention, including ascending aortic replacement and aortic arch repair.

**Figure 10:**
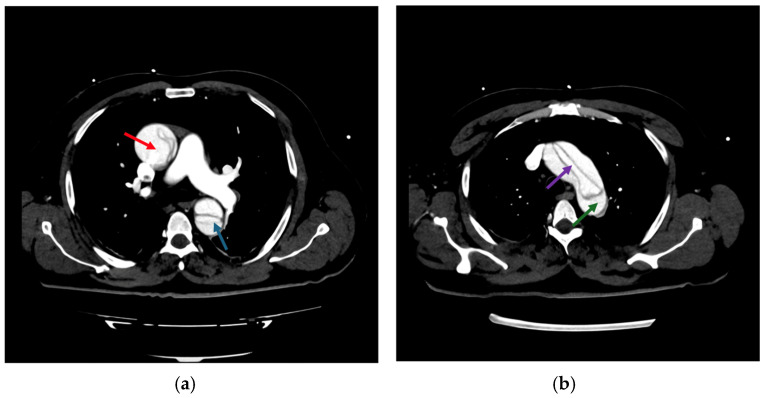
CT imaging of Stanford Type A aortic dissection: true and false lumen analysis—transverse sections: (**a**) compression of the true lumen (purple arrow) by the false lumen (green arrow) within the ascending aorta indicates compromised blood flow, necessitating immediate surgical intervention to restore adequate perfusion; (**b**) involvement of the descending thoracic aorta shows significant expansion of the false lumen (red arrow), with partial thrombosis and dynamic obstruction impacting distal perfusion (blue arrow). These findings underscored the urgency of surgical repair to prevent ischemic complications.

Imaging modalities, especially CTA, were essential for diagnostic and anatomical assessment. In our cohort, CTA findings directly influenced the management pathway in multiple instances. For example, in four cases (15.4%), pericardial effusion detected on imaging necessitated immediate surgical intervention to prevent cardiac tamponade. In nine patients (34.6%), extensive aortic arch involvement observed on CTA dictated the need for total arch replacement. Additionally, imaging findings identified branch vessel involvement in six patients (23.1%), which prompted closer intraoperative vascular assessment to prevent ischemic complications. These findings demonstrate that imaging was not merely a diagnostic tool but also a cornerstone in therapeutic decision-making, shaping both the urgency and extent of surgical intervention. This was exemplified by a representative case of a 57-year-old male with a complex DeBakey Type I dissection (Figure 9 and Figure 10). The case underscores the critical role of advanced imaging in identifying the extent of dissection and guiding timely surgical intervention, which is consistent with our study findings. The detection of aortic regurgitation in 26.9% and pericardial effusion in 19.2% underscores the efficacy of multimodal imaging in identifying problems that require immediate surgical intervention. Outcomes of surgical versus conservative management in our sample reflect findings from IRAD and other registries, revealing in-hospital mortality rates of 12.5% for surgically treated patients compared to 60.0% for those receiving conservative management. The decision between surgical and conservative management was primarily driven by patient stability, comorbidities, and imaging findings. Hemodynamic instability, evidence of rupture, and significant pericardial effusion were immediate surgical indications observed in 61.5% of our cohort. Conversely, conservative management was chosen in patients with advanced age, significant comorbidities, or family refusal of surgical intervention. Despite efforts to stabilize conservatively managed patients with pharmacological blood pressure control and vigilant monitoring, outcomes remained significantly inferior, highlighting the limitations of non-surgical approaches in Stanford Type A dissections. Kaplan–Meier survival studies indicated an 87.5% one-year survival rate for surgical cases against 30% for conservative care, highlighting the critical life-saving effect of prompt surgical intervention [20,21].

Postoperative complications, including acute kidney damage (AKI) necessitating CRRT (25%) and prolonged mechanical ventilation (31.3%), underscore the considerable morbidity linked to AAD care. Perioperative management was critical in influencing patient outcomes in our cohort. Preoperatively, pharmacological stabilization with beta-blockers and vasodilators successfully reduced systolic blood pressure to below 120 mmHg in 85% of patients. Intraoperatively, hypothermic circulatory arrest and selective cerebral perfusion were employed in 26.9% of cases to minimize ischemic injury during aortic arch repairs. Postoperatively, CRRT was initiated early in patients with AKI, and prolonged mechanical ventilation (>48 h) was necessary for 31.3% of surgical patients. Early mobilization and targeted respiratory physiotherapy were essential in reducing pulmonary complications and expediting recovery. These perioperative measures collectively contributed to improved survival and reduced morbidity in the surgical cohort. The long-term impact of these complications on quality of life is a topic receiving increasing attention in the medical literature, as seen in other conditions, such as neoplastic diseases, where organ failure significantly influences patient outcomes. Conservative instances demonstrated elevated incidences of visceral ischemia (20%) and heart failure (10%), consistent with findings indicating that postponed surgery or a lack of surgery aggravates systemic consequences. Innovative diagnostic and management procedures provide optimism for minimizing diagnostic delays and problems. Instruments like the Aortic Dissection Detection Risk Score (ADD-RS) and biomarkers (e.g., D-dimer) have demonstrated potential in risk stratification and imaging prioritization. Furthermore, advancements in perioperative management, such as extracorporeal membrane oxygenation (ECMO) for critical respiratory failure and customized anti-inflammatory treatments, underscore the possibility of enhanced outcomes [22,23,24,25,26].

**Figure 11:**
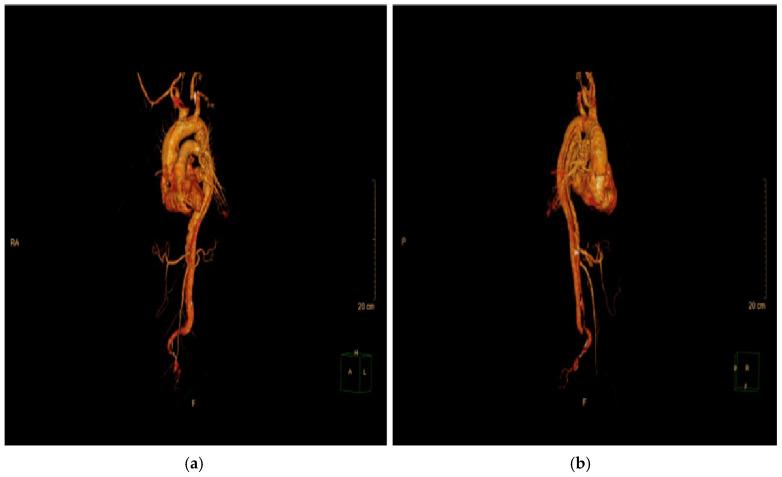
Three-dimensional reconstruction of Stanford Type A aortic dissection—preoperative planning and postoperative assessment: (**a**,**b**) preoperative imaging: the 3D reconstructions display the extensive dissection involving the ascending aorta, aortic arch, and descending thoracic aorta. These images clearly visualize the dissection flap and luminal architecture, aiding in the precise identification of entry and re-entry tears. The preoperative assessment facilitated critical surgical decisions, including the selection of graft size (30 mm Dacron graft) and the extent of aortic arch replacement. Additionally, the reconstructions provided essential insights for planning hypothermic circulatory arrest and cerebral perfusion strategies during surgery.

Advancements in diagnostic imaging have significantly enhanced the early identification and management of acute Stanford Type A aortic dissections. Methods like CTA and ECG-gated scans have markedly shortened diagnostic delays, facilitating accurate and prompt detection of dissections. The hyperacute nature of aortic dissection presents complications, especially in emergency situations where clinical manifestations may resemble those of myocardial infarction or pulmonary embolism. To tackle these challenges, improved imaging methods and algorithms for differential diagnosis are essential, especially in high-volume emergency facilities, to guarantee that the advantages of contemporary diagnostic equipment result in persistent enhancements in patient outcomes [7,22,27]. The contrast between surgical and conservative management outcomes is further illuminated by the illustrative case, where emergency surgical intervention was necessary to address multi-level vascular involvement and preserve organ function. This case highlights the importance of immediate decision-making and multidisciplinary care, aligning with our cohort results (Figure 11).

Hypertension is a significant risk factor in the development of aortic dissections, directly increasing aortic wall stress and causing medial degeneration. This mechanism underscores the influence of elevated blood pressure in triggering the dissection process and its associated complications. In clinical practice, rigorous blood pressure management before surgical intervention has become essential, aiming to reduce complications such as cardiac tamponade and end-organ ischemia. Nevertheless, the interaction between hypertension, dynamic obstruction of the true lumen, and end-organ perfusion remains a considerable challenge in perioperative management. This emphasizes the necessity for a multidisciplinary approach that incorporates acute pharmacological control and long-term strategies to alleviate the effects of hypertension on the aortic wall [21,24,28]. These complications significantly influenced patient prognosis, underlining the importance of multidisciplinary care. A total of 42.3% were either hypotensive or experienced episodes of syncope upon arrival.

The finding of independent mortality factors, including advanced age and hypotension upon presentation, highlights the necessity of personalized treatment in the management of aortic dissections. For senior patients, whose surgical mortality risks are elevated, it is essential to customize therapies to reconcile the advantages of surgery with individual patient vulnerabilities. The involvement of connective tissue disorders in predisposing persons to dissections underscores the necessity for genetic screening and counseling within long-term care regimens. These treatments, along with consistent imaging and monitoring, can reduce the risks of re-dissection or rupture. Effective management strategies that combine surgical intervention with thorough follow-up and individualized care plans are crucial for enhancing survival and quality of life in high-risk patient populations [2,18,29].

CTA has long been established as the gold standard in the diagnosis and evaluation of AADs, owing to its unparalleled ability to provide detailed, rapid, and accurate anatomical visualization of vascular structures. Its widespread adoption across medical centers globally reflects its critical role in modern vascular imaging. This study adheres to these well-recognized diagnostic practices, emphasizing the utility of CTA not as a novel tool, but as an indispensable part of routine clinical evaluation for suspected aortic dissections. While recent advancements in imaging technology have enhanced the resolution and efficiency of CTA, its application in this study aligns with standard protocols for the comprehensive assessment of dissection extent, true and false lumen identification, and preoperative planning. Our findings underscore the universal value of this diagnostic modality, reinforcing its essential role in guiding timely and effective surgical and medical interventions. By adhering to these established practices, our work contributes to the broader body of evidence supporting the continued reliance on CTA as a cornerstone of aortic dissection management.

Despite the valuable insights provided by our study, several limitations must be acknowledged. The relatively small sample size of 26 patients and the single-center, retrospective design may have limited the generalizability and introduced potential selection and reporting biases. Additionally, the study assessed only one-year survival outcomes, lacking longer-term follow-up data to evaluate chronic complications. Variability in treatment approaches, particularly among conservatively managed patients, and slight differences in imaging techniques and surgical methods over the study period may have also introduced confounding factors. Future research should aim to address these limitations through larger, multicenter studies with standardized protocols and extended follow-up durations. Another notable limitation is the absence of advanced imaging modalities, such as MRI, in certain cases, which could have provided additional insights into the extent of dissection and end-organ perfusion. Furthermore, variability in postoperative care protocols, particularly in the conservative management group, may have introduced additional confounding factors. Future studies should aim to integrate multimodal imaging approaches and standardized perioperative protocols to further refine management strategies and improve outcomes.

## 5. Conclusions

This analysis elucidates critical factors affecting mortality and duration of hospitalization in patients with aortic dissection, namely Stanford Type A. Timely recognition of high-risk patients, coupled with immediate surgical intervention and thorough treatment, can markedly enhance results. These findings emphasize the necessity of customized strategies in emergency care to enhance patient survival and recovery.

## Figures and Tables

**Table 1:** Clinical characteristics stratified by 30-day survival in Stanford Type A aortic dissection patients.

Characteristic	Survivors (*n* = 18) Mean ± SD/%	Non-Survivors (*n* = 8) Mean ± SD/%	*p*-Value
Age (years)	56.4 ± 7.5	62.3 ± 8.1	0.04
Male gender (%)	61.10%	50.00%	0.45
BMI (kg/m^2^)	26.1 ± 3.1	27.4 ± 2.9	0.32
Systolic BP (mmHg)	132.5 ± 15.3	118.4 ± 20.2	0.02
Diastolic BP (mmHg)	78.3 ± 9.4	72.1 ± 10.8	0.07
Serum creatinine (mg/dL)	1.1 ± 0.4	1.8 ± 0.7	0.03
LVEF (%)	57.8 ± 6.2	49.2 ± 5.8	0.01
Pericardial effusion (%)	16.70%	50.00%	0.02
Wall motion abnormalities (%)	22.20%	62.50%	0.005
Neurologic deficits (%)	11.10%	37.50%	0.04
CPB time (minutes)	92.3 ± 15.6	105.6 ± 18.4	0.06
Time to surgery (hours, only in surgical patients)	10.5 ± 3.2	16.8 ± 5.6	0.03
Preoperative complications (%)	38.90%	75.00%	0.02
Postoperative complications (%)	22.20%	50.00%	0.04

**Table 2:** Cox regression analysis for mortality predictors in Stanford Type A aortic dissection.

Variable	Cox Regression (HR, 95% CI, *p*-Value)
Age (years)	1.12 (1.02–1.24), *p* = 0.03
Admission hypotension	3.30 (1.40–7.75), *p* = 0.005
Treatment modality (surgical vs. conservative)	0.29 (0.11–0.74), *p* = 0.01

**Table 3:** Influence of surgical timing on short- and long-term mortality in Stanford Type A aortic dissection.

Time to Surgery (h)	Number of Patients (*n*)	30-Day Mortality (%)	1-Year Survival (%)	*p*-Value
≤12 h (early surgery)	10	10	90	0.02
>12 h (late surgery)	6	32	71	0.03

## Data Availability

This article does not include any additional primary data besides the information already presented in the case report section.
